# A Pilot Study on the Impact of Cranberry and Ascorbic Acid Supplementation on the Urinary Microbiome of Healthy Women: A Randomized Controlled Trial

**DOI:** 10.3390/antibiotics14030278

**Published:** 2025-03-07

**Authors:** Alina Nussbaumer-Pröll, Bela Hausmann, Maria Weber, Petra Pjevac, David Berry, Markus Zeitlinger

**Affiliations:** 1 Department of Clinical Pharmacology, Medical University of Vienna, 1090 Vienna, Austria; alina.nussbaumer-proell@meduniwien.ac.at (A.N.-P.); maria.weber@meduniwien.ac.at (M.W.); 2 Joint Microbiome Facility (JMF) of the Medical University of Vienna and the University of Vienna, 1090 Vienna, Austria; bela.hausmann@meduniwien.ac.at (B.H.); petra.pjevac@univie.ac.at (P.P.); david.berry@univie.ac.at (D.B.); 3 Department of Laboratory Medicine, Division of Clinical Microbiology, Medical University of Vienna, 1030 Vienna, Austria; 4 Department of Microbiology and Ecosystem Science, Centre for Microbiology and Environmental Systems Science, Division of Microbial Ecology, University of Vienna, 1030 Vienna, Austria

**Keywords:** urinary tract infection, microbiome, proanthocyanidin, ascorbic acid

## Abstract

**Background**: The collection of microorganisms that colonize the human genital and urinary tract is referred to as the genitourinary microbiome. Urinary tract infections (UTIs), which predominantly affect women, are linked to alterations in the genitourinary microbiome. Cranberries (*Vaccinium oxycoccos*), rich in proanthocyanidins, and ascorbic acid (vitamin C), known for their urinary acidification properties, are commonly used for UTI prevention. However, their effects on the genitourinary microbiome remain inadequately characterized. This pilot study assesses the genitourinary microbiome composition in healthy women and evaluates the influence of cranberry and ascorbic acid supplementation. **Methods**: In a randomized, controlled, and open-label trial, 27 healthy women in their reproductive age (18–40 years) were assigned to three groups: cranberry (n = 8), ascorbic acid (n = 10), and control (n = 9). Urine samples were collected at three time points and processed for 16S rRNA gene amplicon-based microbial community composition analysis. Microbiome composition was compared within and between groups, and between study visits. **Results**: Sufficient microbial DNA was extracted from all midstream urine samples. The genitourinary microbiome was predominantly composed of *Lactobacillus* spp., as reported previously. No significant shifts in microbial composition were observed in response to cranberry or ascorbic acid supplementation, and no statistically significant differences were detected between the intervention and control groups or between study visits. **Conclusion**: The genitourinary microbiome of healthy women remained stable during cranberry or ascorbic acid supplementation. Further studies in patients with recurrent UTIs are needed to explore the potential impacts of these supplements on the genitourinary microbiome in disease states.

## 1. Introduction

Various human body sites harbor many microbiota, with their collective genomic material known as the microbiome [1]. These microbial communities are integral to host physiology, contributing to metabolism, nutrient absorption, energy homeostasis, and immune modulation [2,3].

The urogenital tract is recognized as hosting a distinct and specific microbiome. Advanced molecular techniques, including 16S rRNA gene amplicon sequencing and expanded quantitative urine cultures (EQUC), have identified commensal bacterial populations in the urine of healthy individuals, leading to the conceptualization of the urinary microbiome, or urobiome [4,5]. This microbiome is increasingly implicated in urological health and disease, particularly in conditions like urinary tract infections (UTIs), urgency urinary incontinence (UUI), and interstitial cystitis (IC) [6,7]. Notably, *Lactobacillus* spp. have been associated with lower urinary tract health, while deviations in their abundance correlate with urological pathologies, including IC and UUI [8,9].

Cranberry (*Vaccinium oxycoccos*) products, which are rich in proanthocyanidins (PACs), particularly A-type PACs, as well as flavonols, anthocyanins, organic acids, and benzoic acid, are thought to prevent the adhesion of uropathogenic *Escherichia coli* to urinary epithelial cells [10,11]. However, evidence of their efficacy remains inconclusive. A Cochrane review found no significant reduction in UTI incidence compared to controls when larger studies were considered, though subgroup analyses suggested potential benefits for women with recurrent UTIs [12]. Subsequent meta-analyses indicated a 26% reduction in recurrent UTIs among healthy women and noted benefits for those undergoing gynecological surgery, but emphasized the need for robust large-scale studies to confirm these findings [13,14].

Vitamin C (ascorbic acid) is another potential preventive agent, thought to acidify urine and enhance the conversion of nitrate to nitrogen oxides, inhibiting bacterial growth [15]. Evidence for its efficacy is limited, with mixed outcomes in small studies. While one study in pregnant women suggested a reduction in UTI rates [16], others, including a trial in neuropathic bladder patients, showed no significant effects [17]. A pilot study combining vitamin C, cranberries, and *Lactobacillus rhamnosus* supplementation found promising results, with 72% of women experiencing reduced UTI recurrence at three months [18].

Given the limited understanding of the effects of prophylactic treatments with cranberry and vitamin C on recurrent UTIs and the urinary microbiome, this randomized, controlled, and open-label pilot study aimed to characterize the urinary microbiome of healthy premenopausal women before and after a 10-day regimen of cranberry toffees or ascorbic acid capsules. The combination of cranberry and vitamin C is often recommended to patients with UTIs, as the acidification of urine is thought to enhance the full effect of cranberry PACs in UTI prophylaxis.

## 2. Results

### 2.1. Study Aim and Sample Analysis

This study aimed to evaluate the potential impact of cranberry and ascorbic acid supplements on the genitourinary microbiome of healthy women. Only samples from participants who completed all three visits, submitting at least 150 mL of urine per visit, were included for analysis. Participants who discontinued (*n* = 3) due to restrictions during the COVID-19 pandemic were excluded. A summary of the characteristics of the study participants is given in Table 1.

### 2.2. Demographics

A total of 27 women aged 19–38 participated, with a mean age of 26. Participants were randomly assigned to one of three groups: cranberry (n = 8), ascorbic acid (n = 10), or control/non-intervention (n = 9). The majority of participants (75%) were aged 20–29, with 45% falling into the 20–24 age range. The cranberry and control groups had a mean age of 26, while the ascorbic acid group had a mean age of 25.

One-third of participants used contraception, predominantly oral contraceptives (77.8%), with the remainder using intrauterine devices. Contraceptive use varied across groups, with one participant in the cranberry group and four participants in each of the other two groups using contraceptives.

### 2.3. Volume Intake

Participants in the intervention groups took supplements for 10 days, during which all participants, including those in the control group, documented their daily fluid intake. Two documentation inconsistencies were addressed: Subject PS01 reported drinking 3.5–4 L on Day 1 and 3–4 L on Day 10, for which averages were calculated. Subject PS17 recorded a daily intake of less than 2 L on two occasions, which was specified as 1.5 L. The majority (75%) of participants reported drinking between 1.5 and 2.5 L of water and other beverages per day.

### 2.4. Timing of Urine Sampling

Urine samples were collected between 8 a.m. and 4 p.m., and were divided into three intervals: morning (8:00–10:39 a.m.), noon (10:40 a.m.–1:19 p.m.), and afternoon (1:20–4:00 p.m.). Most samples (55.6%) were collected at noon, with 34.6% in the afternoon and 9.9% in the morning.

### 2.5. Urinary pH

Urinary pH was measured five times per participant, including three clinic visits and two self-administered dipstick tests on Day 5 and Day 10 of supplement intake. At baseline, the mean pH across all groups was 5, which increased to 6 during the study. The overall mean pH across all groups was 6, consistent with physiological levels.

In the cranberry group, pH remained stable at 6 throughout the study. The ascorbic acid group had a lower baseline pH of 5, which increased to 6 by the third measurement, contrary to the expected acidification effect. The control group initially had a mean pH of 6, which dipped to 5 on Day 5 and Day 10 before returning to 6. Isolated pH spikes of 8 were observed in six participants at different time points across all groups, but they did not significantly affect group averages.

### 2.6. Medication Use

Paracetamol was allowed throughout the study and was used by eight participants, primarily for headaches (66.7%) or menstrual pain (33.3%). One participant with enteritis took additional medications, including Imodium^®^ (loperamide), Iberogast^®^, and charcoal tablets, but remained in the study as no interference was assumed.

### 2.7. Adverse Events

A total of 17 adverse events (AEs) were recorded, with 15 documented and 2 described as headaches in participant diaries. The ascorbic acid group accounted for 60% of all AEs. The most frequent complaints were headaches/migraines (53.3%), menstrual pain/disorders (20%), and gastrointestinal symptoms (20%). Only one subject reported cold symptoms (sore throat, cough). All AEs were mild or moderate in severity, resolved by the study’s end, and deemed unrelated to the study supplements in 80% of cases, with one assessed as possibly related.

### 2.8. Feasibility of Urine Sample Processing for Microbiome Analysis

The processing of midstream urine sample pellets for microbiome analysis was successful, yielding sufficient microbial DNA for marker gene amplification and sequencing for most specimens. However, the samples from participant PS19 had insufficient microbial biomass, and one sample (V3) from PS126 was excluded due to sample contamination during handling.

### 2.9. Taxonomic Overview

**Figure 1:**
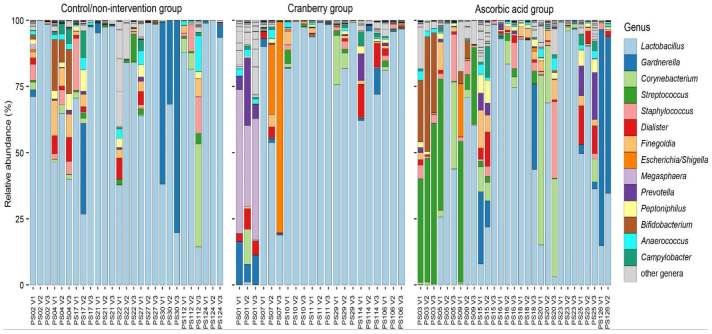
Microbial diversity detected in midstream urine samples, conglomerated on the genus level. The relative abundance of the 14 most frequently occurring genera across all samples is depicted in colored bars, while the remaining genera are depicted in gray as “other genera”. Participant (PS) and visit (V) identifiers are plotted on the x-axis, and participant data are split by intervention groups. Samples from PS19 (cranberry group) were excluded due to low yield, and the Visit 3 sample from PS126 (ascorbic acid group) was excluded due to handling contamination.

Across all analyzed samples, ASVs related to five phyla (*Actinobacteriota*, *Bacteroidota*, *Campylobacterota*, *Firmicutes*, and *Proteobacteria*) were detected. Most samples were dominated by *Lactobacillus* spp., consistent with the microbiome of the healthy vaginal flora (Figure 1). Samples from two participants were dominated by *Gardnerella* spp., which were also detected in 17 other participants, albeit at much lower relative abundances (Figure 1). Furthermore, ASVs related to gut-associated microbes (e.g., *Enterobacteriaceae* spp., *Prevotella* spp., and *Bifidobacterium* spp.) and skin flora (e.g., *Corynebacterium* spp. and *Staphylococcus* spp.) reflected potential carryover from other body sites, known to occur during midstream urine sampling.

### 2.10. Stability and Variability of Microbiome Composition

The microbial composition of samples remained stable within individuals across visits (Figure 1). Changes in microbiome composition were observed in a few participants (PS07, PS09, PS15, PS18), but appeared nonsystematic. In detail, PS07 (cranberry group) had an increase in *Enterobacteriaceae* spp. in Visits 2 and 3, corresponding to a decrease in *Lactobacillus* spp. The urine sample collected from PS07 from Visit 3 contained 75 leukocytes/μL, which was suggestive of a possible urinary tract infection, although no symptoms were reported. For PS09 (ascorbic acid group), *Enterobacteriaceae* spp. were detected only in Visit 1, prior to supplementation, and not in follow-up visits. In this participant, despite ascorbic acid intake, the urine pH remained consistently at 5.

**Figure 2:**
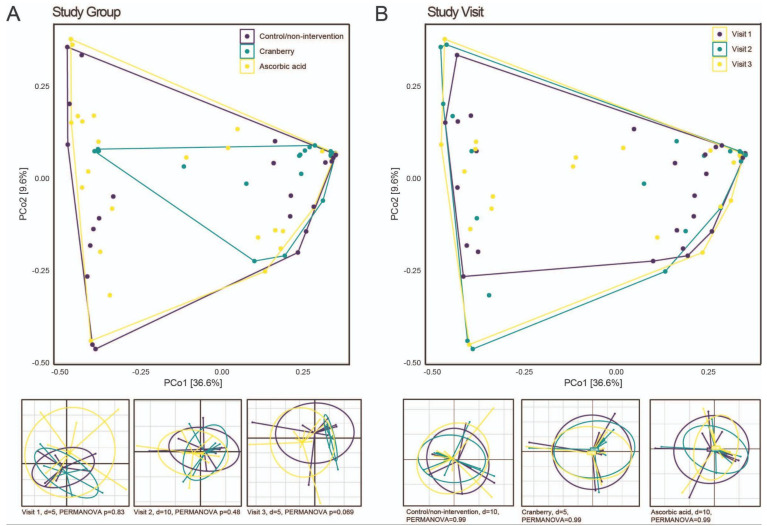
(**A**) Beta diversity between the study groups overall (**top**) and separated by study visits (**bottom**). (**B**) Beta diversity between the study visits overall (**top**) and separated by study group (**bottom**).

Overall, shifts in microbiome composition occurred only in a few patients, with no consistent patterns based on treatment or visit observed. When comparing community composition between study groups (Figure 2A) or between visits (Figure 2B), either across all samples or resolved by visit or study group, respectively, no specific sampling time points or treatments were detected (Figure 2).

## 3. Discussion

The genitourinary microbial community composition data of healthy females collected in this study align with the existing literature, with Lactobacillus consistently detected as the relatively most abundant genus in midstream urine samples [19]. The similarity of midstream urine samples to vaginal samples can be attributable to the anatomical proximity of the urethra and vagina, facilitating bacterial transfer during urination, but also more generally between these body sites. A reduction in the abundance of *Lactobacillus* spp. in the vaginal microbiome has been associated with an increased risk of urogenital infections, including UTIs [20]. Thus, a well-balanced vaginal microbiome may be critical in preventing such conditions. In addition to *Lactobacillus* spp., bacteria typically associated with the human skin and gut microbiome were also detected in the urine samples, likely originating from contamination during midstream urine collection [21].

The microbiome composition was distinct between and predominantly stable across multiple visits for each participant, although variations in the abundance of bacterial genera were observed across all groups. These variations in relative abundances occurred only in a small number of participants, were not statistically significantly related to the interventions, and appeared to occur stochastically. Given these results, we conclude that the interventions did not induce significant changes in genitourinary microbiome composition.

One strength of this study was the implementation of a standardized urine processing protocol, which ensured consistency in sample material subjected to microbiome analysis. This method, involving precise urine collection and centrifugation, generated samples with sufficient microbial biomass for compositional evaluation, and represents a reliable approach for studying genitourinary microbiota. Such a method could be invaluable for future investigations, including those focusing on patients with recurrent UTIs. Additionally, the timing of sample collection post menstruation minimized contamination risks, further standardizing the analysis.

The use of participant diaries enabled the self-monitoring of fluid intake, supplement use, and pH measurements, promoting adherence to study protocols. However, the reliability of self-reported data on fluid intake and supplement adherence remains uncertain and may have influenced the results.

This study also had limitations. The exclusion of postmenopausal women reduces the applicability of the findings to older populations, where *Lactobacillus* spp. are less prevalent, and anaerobic bacteria such as Prevotella and Gardnerella dominate vaginal microbiomes in a higher number of individuals [22]. This vaginal microbiome shift may explain the increased susceptibility to UTIs and bladder disorders in postmenopausal women [23]. Future studies should include postmenopausal participants subjected to the here-tested treatment to explore differences in genitourinary microbiota responses to treatment between reproductive and postmenopausal age groups.

Hormonal contraception use was not controlled, which may have influenced the results. Combined oral contraceptives (COCs) are known to promote Lactobacillus dominance in the vaginal microbiome, whereas intrauterine devices (IUDs) have been linked to a higher risk of bacterial vaginosis in some studies [24]. While no participants changed their contraception during the study, its potential influence on the effect of the tested treatment on the genitourinary microbiome composition should be addressed in future research.

Contamination from skin and perianal bacteria remains a challenge in urinary microbiome studies using midstream urine samples. Alternative collection methods, such as pre-cleaning wipes or urine collection devices, have not reliably mitigated contamination [25,26]. While aseptic methods, such as suprapubic bladder puncture or urethral catheterization, provide more sterile samples, they are invasive and carry risks like infection and discomfort [27]. For healthy individuals, the use of midstream urine collection remains the preferred method due to its non-invasiveness.

This study’s inability to control daily fluid intake may have influenced bacterial concentrations and urine dilution, complicating comparisons between samples. Monitoring fluid intake or standardizing sample collection, such as first-morning urine, could improve reliability in future studies. Dietary habits were not controlled or documented, which may have affected urinary pH measurements. Alkaline urine is associated with diets rich in fruits and vegetables, whereas meat and dairy consumption lowers pH [28,29]. Future research should account for dietary factors to better understand their impact on urinary pH and microbiota.

In addition to cranberry and vitamin C, compounds from garlic (known for its antimicrobial allicin), pomegranate (containing antimicrobial ellagic acid and punicalagins), and berberis (which contains the microbiome-modulating alkaloid berberine) have demonstrated bioactive properties that may influence microbial communities, including those in the urinary tract and/or gut [30,31,32]. However, the impact of these compounds on the microbiome was not the focus of this study.

## 4. Materials and Methods

### 4.1. Ethics

This study involving healthy volunteers was conducted at the Department of Clinical Pharmacology, Medical University of Vienna (Austria), in compliance with the Declaration of Helsinki and the Good Clinical Practice Guidelines of the International Conference on Harmonization. Prior to initiation, the study received approval from the Ethics Committee of the Medical University of Vienna (approval number EK-Nr.1956/2019). All participants provided both oral and written informed consent before being included in the study.

### 4.2. Study Design and Population

This open-label controlled trial was conducted to evaluate the effects of cranberry and ascorbic acid supplementation on the genitourinary microbiome. A total of 30 participants were enrolled and randomly assigned in a 1:1:1 ratio to one of three groups, of whom three discontinued due to restrictions during the COVID-19 pandemic. Consequently, the study included 27 healthy females aged 18–40 years, in one of three groups: cranberry toffees (n = 8), ascorbic acid capsules (n = 10), or control (n = 9). Randomization was achieved using an online block randomization tool [33].

Participants were included if they were healthy women aged 18–40 years, all within their reproductive age, on days 6 to 8 of their menstrual cycle, and had not changed their contraception method within three months prior to or during the study. Individuals were excluded if they had urinary tract anomalies, recent urological or gynecological interventions, relevant cancers, impaired kidney function, leukocyturia (>75 cells/mL), symptoms of urinary tract infection (UTI), or diabetes. Additional exclusion criteria included recent vitamin C supplementation, pregnancy, allergy to the study supplements, or use of concomitant medications, except for contraceptives and paracetamol-based pain medication.

### 4.3. Study Protocol and Procedures

Following an initial phone screening, eligible participants were invited to the clinic for the screening visit (Visit 0, V0) and, if eligible, proceeded to the first study visit (Visit 1, V1), which could occur on the same day. Written informed consent was obtained, and inclusion and exclusion criteria were reassessed. Randomization assigned participants to one of three groups, and baseline urine dipstick tests and pregnancy tests were performed.

**Figure 3:**
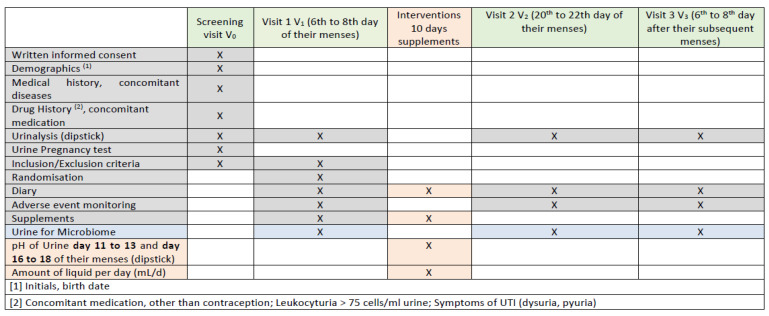
The study schedule shows the indications at each visit during the study period. Gray cells marked with X: Indicate procedures repeated at multiple visits. Blue cells marked with X: Represent urine collection for microbiome analysis. Light orange cells marked with X: Correspond to data collection on urine pH measurements on specific days (days 11–13 and 16–18 of the menstrual cycle), diary checks, and supplement dispensing.

Participants in the intervention groups consumed their respective supplements for 10 days, documenting daily fluid intake, supplement timing, well-being, and urinary pH (using dipstick tests) on specific days of their menstrual cycle. Follow-up visits (V2, days 20–22; V3, days 6–8 of the subsequent menstrual cycle) involved reviewing diaries, documenting adverse events, and collecting final urine samples for microbiome analysis. A study schedule overview is given in Figure 3.

Participants were recruited from a catalog of healthy volunteers who had previously participated in other studies at the study site and were contacted to inquire if they would be interested in taking part in this study. Additionally, flyers providing an overview of the study (without stating the amount of compensation) and contact details were posted in the General Hospital of Vienna and student areas, such as the library, to further advertise the study.

### 4.4. Study Supplements

Participants in the cranberry group consumed Alpinamed^®^, Preiselbeer Direkt (Gebro Pharma GmbH, Fieberbrunn, Austria), delivering 180 mg of proanthocyanidins daily (two toffees, twice daily). The ascorbic acid group received Pure Encapsulations^®^, Vitamin C 1000 (Medico GmbH, Graz, Austria), providing 480 mg of vitamin C daily in a single capsule. The control group received no supplementation.

### 4.5. Urine Sampling, Processing, and Storage

Midstream urine samples were collected at V1, V2, and V3. Leukocyturia was checked using dipstick tests (>75 cells/mL). A 150 mL aliquot was centrifuged at 4000× *g* for 30 min at 4 °C, and the pellet was snap-frozen at −20 °C before storage at −80 °C. Samples were transferred to the Joint Microbiome Facility (JMF) of the Medical University of Vienna and the University of Vienna for microbiome analysis.

### 4.6. Microbiome Analysis

DNA extraction from urine pellets was performed at the JMF (project ID JMF-2004-2) using the QIAamp DNA Microbiome Kit. The composition of the microbial community was determined by 16S rRNA gene amplicon sequencing, using primers 341F/785R [34] to amplify the V3-V4 hypervariable region of the 16S rRNA gene of most bacteria. Amplified regions were barcoded, multiplexed, and sequenced on the Illumina MiSeq platform, as described previously [34]. Quality-filtered and demultiplexed sequences were analyzed using the DADA2 package for amplicon sequence variant (ASV) inference, applying the recommended workflow [35]. FASTQ reads 1 and 2 were trimmed at 230 with allowed expected errors of 4 and 6, respectively. Taxonomic classification was performed using SINA, in conjunction with the SILVA SSU Ref NR 99 rRNA database (SSU rRNA gene sequences classified using SINA version 1.6.1) [36,37]. Differences in microbiome composition between the treatment groups and samples grouped by visit were tested using PERMANOVA and GLMM-MiRKAT.

## 5. Conclusions

In conclusion, while this pilot study did not detect significant microbiome changes following cranberry or ascorbic acid supplementation, it validated a robust method for urine sample processing. Future research should expand to include postmenopausal women, account for factors like contraception, fluid intake, and diet, and consider aseptic sampling in specific populations. Moreover, exploring these interventions in patients with recurrent UTIs could provide greater insight into their impact in a clinical context.

## Figures and Tables

**Table 1:** The table summarizes the characteristics of the study participants and data collected during the trial. The “Total” column consolidates data for all subjects (*n* = 27), while the subsequent columns present data specific to each study group: cranberry (*n* = 8), ascorbic acid (*n* = 10), and control/non-intervention (*n* = 9). Standard deviations (SD) are provided for relevant numerical data. Percentages in parentheses within the “Total” column sum to 100% vertically for each characteristic, representing the proportion of all participants. In contrast, percentages in the study group columns sum to 100% horizontally for each characteristic, reflecting the distribution within each group.

Characteristics of the Study Participants and Collected Data	Total	Cranberry Group	Ascorbic Acid Group	Control/ Non-Intervention Group
n	27	8	10	9
mean age|SD	26|5.506	26|5.743	25|4.784	26|5.925
contraception				
oral contraceptives	7 (77.8%)	1 (14.3%)	3 (42.9%)	3 (42.9%)
intrauterine device	2 (22.2%)	-	1 (50%)	1 (50%)
mean amount of liquid per day in liters|SD	2.3|0.653	2.6|0.694	2.1|0.404	2.1|0.731
time of sampling				
morning (08:00–10:39)	8 (9.9%)	3 (37.5%)	3 (37.5%)	2 (25.0%)
noon (10:40–13:19)	45 (55.6%)	11 (24.4%)	15 (33.3%)	19 (42.2%)
afternoon (13:20–16:00)	28 (34.6%)	10 (35.7%)	12 (42.9%)	6 (21.4%)
mean pH|SD	6|0.211	6|0.195	6|0.279	6|0.354
intake of medicine				
paracetamol	8 (88.9%)	2 (25.0%)	3 (37.5%)	3 (37.5%)
other	1 (11.1%)	1 (100%)	-	-
reason for intake of paracetamol				
headache/migraine	6 (66.7%)	2 (33.3%)	3 (50.0%)	1 (16.7%)
menstrual pain/disorder	3 (33.3%)	-	1 (33.3%)	2 (66.7%)
adverse event (AE)	17	4 (23.5%)	10 (58.8%)	3 (17.6%)
documented	15 (88.2%)	3 (20.0%)	9 (60.0%)	3 (20.0%)
undocumented	2 (11.8%)	1 (50.0%)	1 (50.0%)	-
kind of AE				
headache/migraine	8 (53.3%)	2 (25.0%)	5 (62.5%)	1 (12.5%)
menstrual pain/disorder	3 (20.0%)	-	1 (33.3%)	2 (66.7%)
gastrointestinal	3 (20.0%)	2 (66.7%)	1 (33.3%)	-
symptoms of a cold	1 (6.7%)	-	1 (100%)	-
severity of documented AE				
mild	8 (53.3%)	2 (25.0%)	6 (75.0%)	-
moderate	7 (46.7%)	1 (14.3%)	3 (42.9%)	3 (42.9%)
relation to study drug of documented AE				
unrelated	12 (80%)	2 (16.7%)	7 (58.3%)	3 (25.0%)
unlikely	2 (13.3%)	-	2 (100%)	-
possible	1 (6.7%)	1 (100%)	-	-

## Data Availability

The 16S rRNA gene amplicon sequencing datasets supporting the conclusions of this article are available in the NCBI repository under BioProject accession number PRJNA1217344.
